# Exploring treatment decision-making in chronic myeloid leukemia in chronic phase

**DOI:** 10.3389/fonc.2024.1369246

**Published:** 2024-07-01

**Authors:** David Andorsky, Vamsi Kota, Kendra Sweet

**Affiliations:** ^1^ Rocky Mountain Cancer Centers, Boulder, CO, United States; ^2^ Department of Medicine: Hematology and Oncology, Georgia Cancer Center, Augusta, GA, United States; ^3^ Department of Malignant hematology, Moffitt Cancer Center, Tampa, FL, United States

**Keywords:** CML treatment, chronic phase, clinical decision-making, expert review, treatment selection, treatment sequencing

## Abstract

The introduction of tyrosine kinase inhibitors (TKIs) has transformed the treatment of chronic myeloid leukemia (CML). Each approved TKI has its own risk-benefit profile, and patients have choices across lines of therapy. Identifying the initial and subsequent treatment that will lead to the best possible outcome for individual patients is challenging. In this review, we summarize data for each approved TKI across lines of therapy in patients with CML in chronic phase, highlighting elements of each agent’s safety and efficacy profile that may impact patient selection, and provide insights into individualized treatment sequencing decision-making aimed at optimizing patient outcomes.

## 1 Introduction

Approximately 65,800 patients were diagnosed with chronic myeloid leukemia (CML) in 2019 (1). The management of CML in chronic phase (CML-CP) has changed significantly since the availability of BCR::ABL1-targeted tyrosine kinase inhibitors (TKIs). These drugs have greatly improved response rates, reduced the risk of disease progression, and dramatically improved long-term survival (2, 3). Patients with CML-CP receiving a TKI have a 5-year survival rate that is only slightly lower than that of the general population and may achieve a nearly normal life expectancy. Despite these advances, it is estimated that 29,930 people worldwide died of CML in 2019 (1).

The current standard of care for patients with CML-CP is therapy with BCR::ABL1-targeted TKIs with the potential for hematopoietic cell transplant (HCT) for eligible patients who no longer respond to these agents or whose disease progresses to a more advanced stage (**Table 1**) (2, 3). Patients newly diagnosed with CML-CP are treated with one of the four TKIs (first-generation TKI imatinib and second-generation [2G] TKIs nilotinib, dasatinib, and bosutinib) approved by the US Food and Drug Administration (FDA) for first-line (1L) use (2, 3). Additional TKIs (i.e., asciminib and ponatinib) are FDA approved for patients in later lines of therapy or who develop CML with the acquired T315I mutation, which predicts resistance to the four TKIs approved in 1L (2, 3).

**Table 1:** Sequential use of BCR::ABL1-targeted TKIs is the current standard of care for patients with CML-CP (2, 3).

	1L	2L	≥3L	T315I
**NCCN**	**Low-risk score**^a^ Preferred regimens: 1G TKI (imatinib); or, 2G TKI (bosutinib, dasatinib, or nilotinib) **Intermediate- or high-risk score** Preferred regimens: 2G TKI (bosutinib, dasatinib, or nilotinib); Other recommended regimen: 1G TKI (imatinib)	**Switch TKI**^b^ **and evaluate for allo-HCT** if *BCR::ABL1* ^IS^ is >10% at 6 or 12 mo **Switch TKI or continue same TKI (other than imatinib) and consider allo-HCT evaluation** if *BCR::ABL1* ^IS^ is >1% to 10% at 12 mo or >10% at 3 mo	**Same as for 2L** **Consider:** (bullet) Ponatinib, asciminib, or omacetaxine for patients resistant to or intolerant of ≥2 TKIs	Ponatinib (preferred) Asciminib Omacetaxine Allo-HCT
**ELN**	Any approved TKI	**Switch TKI**^b^ and evaluate mutations for treatment failure or resistance **Consider:** • Patient and physician choice and options for supportive care for intolerance • Patient factors (e.g., age, comorbidities, AEs with 1L TKI) for suboptimal response	**Consider:** • Sensitivity to specific TKIs after mutational analysis • Allo-HCT in patients with suboptimal response to ≥2 TKIs	Ponatinib

1G, first generation; 1L, first line; 2G, second generation; 2L, second line; 3L, third line; AE, adverse event; allo-HCT, allogeneic hematopoietic cell transplant; CML-CP, chronic myeloid leukemia in chronic phase; ELN, European LeukemiaNet; ELTS, EUTOS long-term survival; IS, International Scale; NCCN, National Comprehensive Cancer Network; TKI, tyrosine kinase inhibitor.

a Based on Sokal, Euro, or ELTS score.

b Switch recommendations based on response to prior TKI as assessed by *BCR::ABL1* ^IS^ level.

Although most patients have a favorable outcome on 1L therapy, between 28% and 64% of patients discontinue 1L therapy within 10 years, mainly due to suboptimal response, loss of response, or treatment intolerance. An assessment of pivotal 1L studies shows that at least half of patients did not meet the goal of major molecular response (MMR) at 1 year, and 5% to 24% discontinued treatment due to an adverse event (AE) (**Table 2**). This suggests that at least half of patients will require another line of therapy. Sequential treatment with different TKIs may be valuable in maintaining favorable long-term outcomes for patients (11). However, with each line of TKI treatment, the rate of treatment failure increases, and treatment durability and long-term survival decrease (12–14). Therefore, careful consideration of the 1L TKI and decisions around treatment sequencing are critical to optimizing each patient’s outcome.

**Table 2:** Comparison of pivotal 1L trials (4–10).

	IRIS Imatinib (n=553)	ENESTnd Nilotinib 300/400 mg (n=282/281)	DASISION Dasatinib (n=259)	BFORE Bosutinib (n=246)
Study design				
**Follow-up, median, y**	10.9	10	5	5
**N**	1106	846	519	536
**Comparators**	Imatinib 400 mg QD vs IFNα + cytarabine	Nilotinib 300 or 400 mg BID vs imatinib 400 mg QD	Dasatinib 100 mg QD vs imatinib 400 mg QD	Bosutinib 400 mg QD vs imatinib 400 mg QD
Molecular milestone, %				
**3-month EMR (*BCR::ABL* ^IS^ ≤10%)**	NR	90.7/89.2	84	75.2
**12-month CCyR (*BCR::ABL* ^IS^ ≤1%)**	52.8	80/78	85	77.2
**12-month MMR (*BCR::ABL* ^IS^ ≤0.1%)**	27.7	44/43	46	47.2
**5-year MR^4.5^ (*BCR::ABL* ^IS^ ≤0.0032%)**	23.0	53.5/52.3	42	47.4
Survival or progression endpoint, % (time point)				
**EFS or PFS**	79.6 (10-y EFS)	86.2/89.9 (10-y PFS)	85 (5-y PFS)	93.3 (5-y EFS)
**OS**	83.3 (10 y)	87.6/90.3 (10 y)	91 (5 y)	94.5 (5 y)
**Progression to CML-AP/BP**	6.9 (10 y)	4.1/2.2 (10 y)	4.6 (5 y)	2.4 (5 y)
Discontinued treatment, %				
**Due to AEs**	6.9	5/9	16	25.4

AE, adverse event; AP, accelerated phase; BID, twice daily; BP, blast phase; CCyR, complete cytogenetic response; CML, chronic myeloid leukemia; EFS, event-free survival; EMR, early molecular response (*BCR::ABL1* ^IS^ ≤10% at 3 months); IFNα, interferon alpha; IS, International Scale; MMR, major molecular response; MR^4.5^, *BCR::ABL1* ^IS^ ≤0.0032%; NR, not reported; OS, overall survival; PFS, progression-free survival; QD, once daily.

The purpose of this review is to examine the current data from pivotal trials for available TKIs across lines of therapy; the rates and most common reasons for treatment discontinuation; and patient and disease variables that influence treatment selection according to these studies, current treatment guidelines, and real-world experience treating patients with CML.

## 2 Treatment of newly diagnosed CML

The four TKIs approved as 1L therapy have been studied extensively in patients with CML-CP, and the safety and efficacy profiles of each have been well established (**Table 2**). These TKIs work by binding the ATP-binding site on the BCR::ABL1 fusion protein, which inhibits the enzymatic activity of the protein, thus inhibiting proliferation and inducing apoptosis in BCR::ABL-positive CML (15). Nilotinib, dasatinib, and bosutinib are 2G TKIs approved for 1L use, each with long-term follow-up data available from pivotal phase 3 trials, ENESTnd, DASISION, and BFORE, respectively.

**Figure 1:**
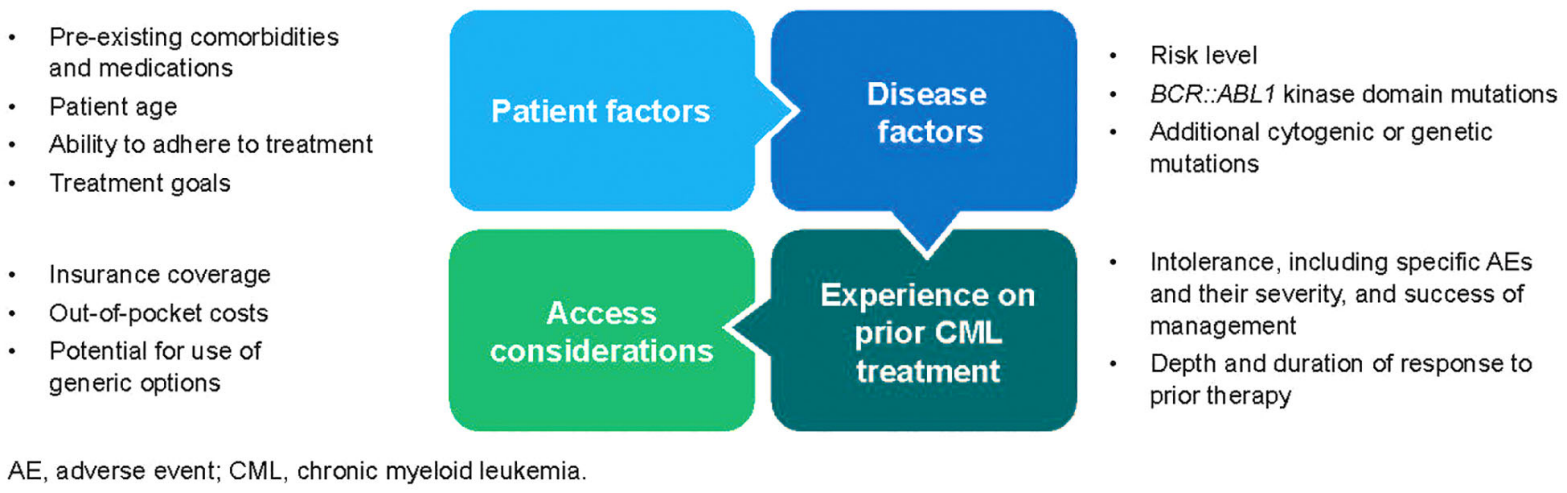
Considerations for treatment selection and sequencing (2, 3). Various patient and disease factors play a role in treatment decision-making across lines of therapy. In addition, a patient’s experience on prior CML treatment, if applicable, along with access and cost considerations must be taken into account when treatment planning.

Overall, 2G TKIs confer significantly higher molecular response rates than imatinib, and while there are several class-related toxicities, each agent has its own unique toxicity profile that must be considered when deciding on a 1L treatment. Decisions regarding 1L treatment selection are based on risk score, comorbidities, potential toxicities and drug interactions, and patient preferences (**Figure 1**) (2, 3). Although National Comprehensive Cancer Network (NCCN) Guidelines state that 2G TKIs are preferred as 1L treatment for patients at intermediate to high risk of disease progression based on risk score, other current treatment guidelines do not provide specific recommendations on which of the four approved therapies should be used first (2, 3). Therefore, physicians must leverage their clinical experience to choose a 1L therapy based on multiple factors, including prognostic risk, disease and patient characteristics (e.g., goals, comorbidities, concomitant medications, insurance coverage, out-of-pocket costs, and ability to adhere to treatment), and cytogenetics (**Figure 1**) (2, 3). Decision-making is further complicated by the lack of biomarkers to help predict response or intolerance.

Patients with high-risk disease and other disease-specific factors that might indicate a risk for worse outcomes are difficult to treat (2, 3). Risk stratification systems are used inconsistently both across clinical trials and in clinics; so, risk scores lack concordance and show differences when predicting outcomes (16). Likewise, in people with and without CML, the incidence of comorbidities and number of concomitant medications increase with age, along with the risk of developing AEs (17).

In general, imatinib remains a preferred treatment option for older patients, those with low-risk disease, or those with significant comorbidities (2, 3). However, the use of imatinib is decreasing, particularly in high-income countries (18). In the US, the use of 1L imatinib has decreased over time, so that >50% of patients receive a 2G TKI in 1L, even among older patients (4–7, 19, 20). This change has been driven by numerous studies that have shown that 2G TKIs are significantly more effective than imatinib in achieving the endpoints of complete cytogenetic response (CCyR) and MMR and can help more patients meet their response milestones and attempt treatment-free remission (TFR), if it is desired and the patient is eligible (**Table 2**). Although some studies suggest that generic imatinib is more cost-effective, the improved efficacy and higher likelihood of achieving TFR with 2G TKIs may lead to overall savings long-term (21).

High-dose imatinib may be an alternative to 2G TKIs because studies suggest that higher doses may be able to overcome some cases of primary resistance; however, no studies have directly compared high-dose imatinib with 2G TKIs. A portion of patients also have mutations and cytogenetic alterations at diagnosis, which could affect treatment selection and outcomes (2, 3). While there are recommendations to manage patients who develop specific resistance mutations while on treatment, less is known about how baseline mutations may affect long-term outcomes or treatment selection (22, 23). Some evidence suggests that additional cytogenetic abnormalities are associated with worse prognosis but not with the likelihood of response to one TKI over another (24–26). Current risk stratification systems do not take these factors into consideration, and in the absence of more data on this topic, physicians need to draw on their expertise to determine how to take these parameters into account when making a treatment selection (27–29).

## 3 Treatment goals and molecular monitoring guidelines

The main goals of therapy are to prolong survival, prevent disease progression to accelerated- or blast-phase CML, achieve response milestones, improve or maintain quality of life (QOL), minimize treatment-related toxicities, and offer eligible patients the opportunity to attempt TFR (2, 3, 30, 31). Individual treatment goals may evolve over time and across lines of therapy but are based on patient- and disease-specific characteristics and each patient’s wishes (2, 3).

Current European LeukemiaNet recommendations and NCCN Guidelines have established similar recommendations for molecular monitoring and assessing treatment efficacy (**Table 3**), with the goal of helping physicians assess the efficacy of current treatments and guide decision-making if a switch is warranted (2, 3). Currently, molecular monitoring is done using blood-based quantitative polymerase chain reaction, and the results of this method strongly correlate with levels of *BCR::ABL1* transcripts in the bone marrow without requiring bone marrow aspirations (3). Molecular monitoring using quantitative polymerase chain reaction is reported using the International Scale (IS), which is expressed as a log-reduction from the standardized 100% (3). Thus, a ≥2-log reduction (*BCR::ABL1* ^IS^ ≤1% or MR^2^) correlates with CCyR, and a ≥3-log reduction (*BCR::ABL1* ^IS^ ≤0.1%) is classified as MMR. Deep molecular responses (DMRs) include MR^4^ (*BCR::ABL1* ^IS^ ≤0.01%) and MR^4.5^ (*BCR::ABL1* ^IS^ ≤0.0032%) (3).

**Table 3:** Treatment response milestones according to guidelines by *BCR::ABL1* ^IS^ levels (2, 3)^a^.

	Response category	Months after treatment initiation			Any time	Actions
		3	6	12		
**NCCN**	Green (TKI sensitive)	≤10%	≤10%	<1%^b^	N/A	• Monitor response and AEs • Continue on the same TKI^b^
	Yellow (possible TKI resistance)	>10%	N/A	>1% to 10%		
						• Evaluate compliance and drug interactions • Consider mutational and bone marrow cytogenetic analyses to assess for MCyR at 3 months or CCyR at 12 months • Switch TKI, continue the same TKI (other than imatinib) • Consider evaluation for HCT
						• Evaluate compliance and drug interactions • Consider mutational analyses • Switch TKI and evaluate for HCT
	Red (TKI resistance)	N/A	>10%	>10%		
**ELN**	**Optimal**	**≤10%**	**<1%**	**≤0.1%**	**≤0.1%**	• **Continue treatment**
	Warning	>10%	>1% to 10%	>0.1% to 1%	>0.1% to 1%, loss of ≤0.1% (MMR)	• Carefully consideration for continuation or change, depending on patient characteristics, comorbidities, and tolerance^c^
	Failure	>10%, if confirmed within 1-3 months	>10%	>1%	>1%, resistance mutations, high-risk additional chromosomal abnormalities	• Change treatment

AE, adverse event; CCyR, complete cytogenetic response; ELN, European LeukemiaNet; HCT, hematopoietic cell transplant; IS, International Scale; MCyR, major cytogenetic response; MMR, major molecular response; N/A, not available; NCCN, National Comprehensive Cancer Network; TKI, tyrosine kinase inhibitor.

a Definitions are the same across lines of therapy.

b If treatment goal is long-term survival, ≤1% at 12 months is optimal. If treatment goal is treatment-free remission, ≤0.1% at 12 months is optimal. If response is optimal, continue the same TKI. A nonoptimal response per goals requires shared decision-making with the patient.

c Additional quantitative polymerase chain reaction testing may be indicated if the kinetics of the response are not clear or if toxicity or intolerance cause dose interruptions or reductions.

Key response milestones include complete hematologic response (*BCR::ABL1* ^IS^ ≤10%) at 3 and 6 months and MMR (*BCR::ABL1* ^IS^ ≤0.1%) within 12 months of initiating therapy (2, 3). In general, the depth (e.g., DMR), timing (e.g., early molecular response [EMR], *BCR::ABL1* ^IS ^<10% at 3 months), and durability of response correlate with prolonged progression-free survival (PFS) and overall survival (OS). In particular, EMR at 3 and 6 months after 1L TKI initiation is seen as an effective predictor of favorable long-term PFS and OS (3). Achievement of *BCR::ABL1* ^IS^ ≤1% within 12 months after initiation of 1L TKI has been established as a prognostic indicator of long-term survival (32, 33). MMR at 12 months is associated with a very low probability of loss of response and a relatively high likelihood of achieving DMR (2, 3).

## 4 Treatment sequencing considerations

Selection of subsequent lines of therapy is based on many of the same factors that played a role in 1L treatment selection, with an added consideration for the patient’s treatment history, including tolerability issues, depth of response, and length of time on prior therapy (**Figure 1**) (2, 3). When deciding on a second-line (2L) therapy, healthcare practitioners (HCPs) also need to consider whether the change in treatment is necessitated by resistance to or intolerance of 1L therapy. A major reason to improve decisions around treatment sequencing is that the rate of treatment failure with currently available TKIs increases with each line of treatment, and survival is inversely related to line of treatment (12–14). A retrospective review evaluating clinical outcomes in 90 patients with CML-CP who received imatinib as their first TKI, followed by dasatinib or nilotinib in the next line, found that the 8-year OS rate decreased from 83% in patients still receiving 1L imatinib to 22% in patients receiving third-line and later (3L+) TKIs, with 5-year OS rates of 82% and 77% in patients receiving 2L and 3L+ TKIs, respectively (13). High failure rates with 3L TKIs put patients at risk of disease progression and death. Understanding how to optimize treatment sequencing is a substantial unmet educational need for HCPs treating patients with CML-CP because it has the potential to improve long-term patient outcomes and preserve their QOL throughout the course of their disease.

Treatment guidelines include response milestone assessments to provide direction on when a TKI switch is warranted (**Table 3**) (2, 3). The guideline recommendations are clear for patients with TKI-sensitive disease or whose treatment is optimal and for those with TKI-resistant or unresponsive disease. Patients who meet the response milestones should continue with the same dose of TKI, with a reassessment of *BCR::ABL1* transcripts every 3 months until such response milestones are not met (2, 3). Patients with TKI-resistant or unresponsive disease may need to increase their dose (if receiving imatinib), switch to an alternate TKI, or be evaluated for allogeneic HCT (allo-HCT) (2, 3). However, the decision to stop one therapy and switch to another is often not clear-cut, especially for patients receiving 1L treatment (**Table 3**) whose treatment response suggests but does not definitively indicate TKI resistance; for these patients, decisions are subjective and must be based on each patient’s individual clinical situation (2, 3).

### 4.1 Defining treatment resistance

Resistance is a clinical definition, based on when a patient does not meet response milestones. Two main categories of TKI resistance are observed: *de novo* or primary resistance and acquired or secondary resistance (34). With *de novo* or primary resistance, a patient is never able to meet response milestones, while with acquired or secondary resistance, a patient loses the response they had previously achieved.

### 4.2 *De novo* or primary resistance

While the mechanisms of resistance are not yet fully elucidated, they are likely multifactorial and complex (34). Primary resistance may be more likely due to BCR::ABL1-independent mechanisms that involve alternative cell survival pathways operating even when BCR::ABL1 is effectively inhibited by TKIs (35). Thus, across studies, approximately one-third of patients who received imatinib did not achieve a response or ultimately lost response. For instance, **Table 2** shows that in the IRIS trial and other 1L trials in which imatinib was a comparator, approximately one-quarter to half of patients treated with imatinib did not achieve CCyR at 1 year, which is the goal according to CML treatment guidelines, and fewer patients achieved MMR at 1 year or DMR by 5 years (2–9, 20). More patients who received nilotinib, dasatinib, or bosutinib achieved responses across all response milestones. Even so, approximately 25% of patients still required a switch from their initial TKI within the first year of treatment, and up to 50% required a switch by 3.5 years (36, 37). For patients with persistent detectable *BCR::ABL1* transcripts while receiving 1L imatinib, switching to a 2G TKI provided higher DMR rates compared with staying on imatinib and may enable more patients to attempt TFR. However, 50% of patients without MR^4.5^ at the time of switching to a 2G TKI from imatinib still did not achieve MR^4.5^ by 48 months after switching.

### 4.3 Acquired or secondary resistance

The reasons patients lose response to treatment and the mechanisms of acquired or secondary resistance are not fully understood and may be related to *BCR::ABL1*-dependent or-independent mechanisms. For considerations of dose adjustments or TKI switch, current guidelines only address acquired resistance due to *BCR::ABL1*-dependent mechanisms that disrupt kinase inhibition (34). Point mutations in the kinase domain (e.g., T315I) that interfere with the ability of TKIs to bind and inhibit BCR::ABL1 are the most well-known mechanism of acquired resistance and have been identified in up to ≥50% of patients experiencing resistance or disease progression (38–45). These mutations are associated with poor prognosis and a higher risk of disease progression (38, 40, 43–46). T315I is the most common acquired mutation, occurring in 10% to 27% of patients with an acquired *BCR::ABL1* mutation, and it confers resistance to all approved 1L TKIs. Additional mechanisms of resistance are thought to include upregulation of other oncogenic cellular pathways or activation of cellular or biological processes that disrupt TKI cellular availability or concentration (35). Additional molecular and cytogenetic testing, including bone marrow cytogenetics and *BCR::ABL1* kinase domain mutational analysis, is recommended for patients not meeting response milestones to determine whether other factors are playing a role in treatment resistance and to guide next steps. However, the therapeutic implications of other resistance mechanisms are not fully understood and are being explored in ongoing research (2, 3).

### 4.4 Sequencing considerations for patients with treatment resistance

In the absence of clear *BCR::ABL1* kinase-domain mutations to guide the decision on 2L treatment, no 2G TKI appears more efficacious than others, so the choice must be almost entirely driven by patient-specific factors, such as ability to tolerate prior TKIs and comorbidities (2, 3). Decisions about maintaining or switching therapy when response milestones are not being met are also highly heterogeneous and complicated. Lack of data from trials and concrete recommendations complicates this decision-making. In the absence of complete treatment failure or lack of response, physicians may try to keep a patient on treatment longer and use dose modifications if possible, particularly with imatinib and if the patient is showing signs of response that are trending in the right direction (3, 39). Some patients may need more time on treatment to achieve a response and meet treatment goals.

For patients receiving imatinib, dose escalation may be an option to overcome primary resistance, especially in patients who had achieved a cytogenetic response but then experienced relapse; however, dose escalation is unlikely to benefit patients who never experienced a cytogenetic response to standard-dose imatinib or were intolerant of high-dose imatinib. Additionally, the responses after dose escalation are reportedly short, and no randomized studies have shown that dose escalation improves PFS in these patients (2, 3). Switching TKIs is therefore recommended for patients whose disease is fully resistant to standard-dose imatinib (3).

Patients who are currently resistant to standard-dose imatinib may benefit from switching to nilotinib, dasatinib, or bosutinib, which are all effective in patients with CML-CP resistant to or intolerant of imatinib (**Figure 1**) (2, 3). Nilotinib and dasatinib have demonstrated higher rates of MMR at 12 months than high-dose imatinib, and achieving EMR after 2L TKI therapy with nilotinib or dasatinib is associated with favorable OS and PFS. Bosutinib has demonstrated activity in the 2L in patients with disease that is resistant to imatinib, nilotinib, or dasatinib (3). Patients with disease that is resistant to nilotinib, dasatinib, or bosutinib may switch to an alternate TKI (except imatinib) or be considered for allo-HCT. Ponatinib is another option for patients in 2L, especially patients who experienced resistance to prior TKIs, since use of another 2G TKI after resistance to a previous 2G TKI has shown limited clinical benefit, and cycling through various TKIs may lead to lower responses during later lines of therapy. However, ponatinib carries a risk of arterial and venous thrombosis and therefore may not be the preferred 2L agent in the absence of a T315I mutation (3).

Without mutations to guide treatment decisions, however, no clear recommendation can be made for an individual TKI (2, 3). Patients with acquired resistance due to the T315I mutation have the option to switch to ponatinib or asciminib, which has been approved for use in the 3L setting and has demonstrated activity in these patients (**Tables 4**, **5**). (1, 3) Ponatinib 45 mg once daily (QD) is the recommended initial dose, but it has been associated with an increased risk of arterial occlusive events; cardiovascular (CV) AEs are highest in patients with pre-existing CV risk factors (such as diabetes, hypertension, hyperlipidemia, smoking, and estrogen use). In the OPTIC study assessing starting doses of 45, 30, and 15 mg QD with dose reductions to 15 mg QD, patients who received ponatinib may have had a decreased incidence of CV events (CVEs) with dose reductions yet maintained a similar response rate (3, 54).

**Table 4:** Summary of pivotal second-line and beyond trials in patients with CML-CP with or without the T315I mutation (47–52).

	Study 200 Bosutinib (n=288)	BYOND Bosutinib (n=144)	CA180-034 Dasatinib (n=167)	Phase 2 Nilotinib (n=294)	PACE (T315I±) Ponatinib (n=449)	X2101 (T315I+) Asciminib (n=48)
Study design						
**Follow-up, median**	2 y	2 y	6 mo	2 y	15 mo	14 mo
**N**	288	163	1158	321	267	141
**Design**	Bosutinib 500 mg QD	Bosutinib 500 mg QD	Dasatinib at various doses (100 mg QD data shown)	Nilotinib 400 mg BID	Ponatinib 45 mg QD	Asciminib 200 mg BID
Molecular milestone, %						
**3-month EMR** **(*BCR::ABL* ^IS^ ≤10%)**	NR	Cumulative CCyR equivalent, 83.7	NR	NR	CCyR equivalent, 54	NR
**12-month CCyR** **(*BCR::ABL* ^IS^ ≤1%)**	41	80.6	41	44	46	70
**12-month MMR** **(*BCR::ABL* ^IS^ ≤0.1%)**	64	71.1	NR	28	44	48
**MR^4.5^** **(*BCR::ABL* ^IS^ ≤0.0032%)**	NR	43.0 at 2 y	NR	NR	NR	24.4 at 96 wk
Survival or progression endpoint, %						
**EFS or PFS**	79 (PFS)	NR	92 (PFS)	NR (EFS)/64 (PFS)	80 (12-mo PFS)	NR
**OS**	92	96.0	98	87	94 at 12 mo	NR
**Progression to ** **CML-AP/BP**	14 at 2 y	0 at 1 y	9 at 6 mo	10 at 24 mo	19 at 12 mo	8 at 14 mo
Discontinued treatment, %						
**Due to AEs**	21	25	4	16	12	8.3

AE, adverse event; AP, accelerated phase; BID, twice daily; BP, blast phase; CCyR, complete cytogenetic response; EFS, event-free survival; EMR, early molecular response (*BCR::ABL1* ^IS^ ≤10% at 3 months); IS, International Scale; MMR, major molecular response; MR^4.5^, *BCR::ABL1* ^IS^ ≤0.0032%; NR, not reported; OS, overall survival; PFS, progression-free survival; QD, once daily.

**Table 5:** Summary of later-line asciminib in patients with CML (53).

Study design	Asciminib ASCEMBL N=233 Asciminib 40 mg BID vs bosutinib 500 mg QD	
**Time point**	**24 wk**	**96 wk**
**CCyR, %**	**40.8**	**39.8**
**MMR, %**	**25.5**	**37.6**
**DMR, %** ** MR^4^** ** MR^4.5^**	**10.8** **8.9**	**17.2** **10.8**
**OS, %**	**97.5 by 1 y**	**97.3 by 2 y**
**Discontinued due to AEs, %**	**5.1**	**7.0**
**Progression to AP or BP**	**NR**	**1**

AE, adverse event; AP, accelerated phase; BID, twice daily; BP, blast phase; CCyR, complete cytogenetic response; DMR, deep molecular response; IS, International Scale; MMR, major molecular response; MR^4^, *BCR::ABL1* ^IS^ ≤0.01%; MR^4.5^, *BCR::ABL1* ^IS^ ≤0.0032%; NR, not reported; OS, overall survival; QD, once daily.

Asciminib is the first BCR::ABL1 inhibitor to Specifically Target the ABL Myristoyl Pocket (STAMP) (55–57). It binds to the myristoyl pocket on the BCR::ABL1 protein, producing a conformational change that inhibits downstream signaling (55–57). This unique mechanism of action may help patients who experienced resistance with TKIs achieve a response. The phase 1 study of asciminib included patients with T315I-mutated CML, and asciminib was approved for use in some countries in this patient population based on these data (**Table 4**) (56).

### 4.5 Intolerance

While NCCN Guidelines lack a standardized definition of intolerance and recommendations for when to switch treatment due to intolerance, evaluating and overcoming intolerance is critical to helping patients stay on treatment (2, 3). Each of the current TKIs has a distinct toxicity profile that plays a critical role in patients’ ability to stay on treatment (2, 3). Improvements in treatment have made CML a chronic disease, requiring lifelong disease management for most patients, and this necessity has made QOL an important treatment goal (2, 3, 30, 31). Some studies and case reports suggest that initiation of TKIs at lower doses or dose reduction may be used to maintain efficacy while minimizing treatment-related AEs (54, 58–60). Treatment-related AEs can lead to a decrease in QOL. In a study evaluating adverse drug reactions experienced by 86 adults with CML-CP receiving imatinib, dasatinib, or nilotinib, more than half of patients reported a decrease in QOL due to AEs (61). In addition, QOL may be worse for patients in later lines of therapy, as their overall health wains and the likelihood of comorbidities increases (2, 3, 62). Therefore, treatment tolerability and maintenance of QOL are essential goals when making treatment decisions.

The criteria for intolerance have varied in clinical trials evaluating TKIs and are usually based on the severity of AEs, evaluated using Common Terminology Criteria for Adverse Events (63). These criteria are designed to identify acute AEs and thus may not account for how persistent low-grade AEs affect patient QOL and treatment adherence (63). Also, individuals differ in their acceptance of various AEs and thresholds for acceptable severities (63). Therefore, intolerance is operationally driven by each individual patient’s experience of AEs that are not resolved using guideline-recommended strategies for prevention and management of treatment-related AEs, including AE-specific dose adjustments and other interventions.

Several class-effect AEs, including edema, gastrointestinal events, skin toxicities, muscle and joint pain, myelosuppression, hepatoxicity, and fatigue, are associated with TKIs (64–67). However, each agent has a unique safety profile with variable incidences and severities of these AEs, along with additional and potentially more serious AEs, such as CV and bleeding complications (nilotinib, ponatinib) and pulmonary complications (dasatinib) (62, 68). These drug-specific AEs are potentially more serious than the class effects and are difficult to predict, particularly in patients with no major underlying comorbidities and also because no biomarkers have been established (2, 3). Many of the class-effect AEs may be less likely to trigger a TKI switch or discontinuation because they tend to be mostly low grade, and physicians often have more experience and guidance on proactive management of AEs common with TKIs and other cancer therapies (2, 3).

Regardless of management strategies, including dose modifications, intolerance remains a primary reason for treatment discontinuation (**Tables 4**, **5**) (8, 47–49, 56). One observational study evaluating TKI use and management patterns in routine clinical practice in Europe and the US found that intolerance is the main reason for switching TKI therapy, emphasizing the need for more tolerable therapies in all lines (69). In the IRIS study of imatinib in 1L, 4% and 6.9% of patients discontinued therapy by 5 and 10 years due to AEs, respectively. In key trials of 2G TKIs used in 1L, discontinuation rates due to AEs ranged from 12.2% to 25.0% by 5 years. Discontinuation rates due to AEs with 2G TKIs and ponatinib increased in later lines of therapy, with 21% to 30% of patients discontinuing due to AEs in 2L+ (**Table 4**) (10, 47, 48, 50, 56).

### 4.6 Addressing intolerance

Assessing patients for potential tolerability issues when selecting a treatment and managing treatment-emergent AEs is an essential part of treatment decision-making (2, 3). Because each TKI has a unique AE profile, switching to another TKI has the potential to mitigate tolerability issues that a patient is experiencing on their current TKI (2, 3). However, physicians should exhaust proactive AE management strategies, including dose adjustments, to alleviate the burden of intolerance prior to initiating a treatment switch to extend the viability of each line of therapy (2, 3).

**Figure 2:**
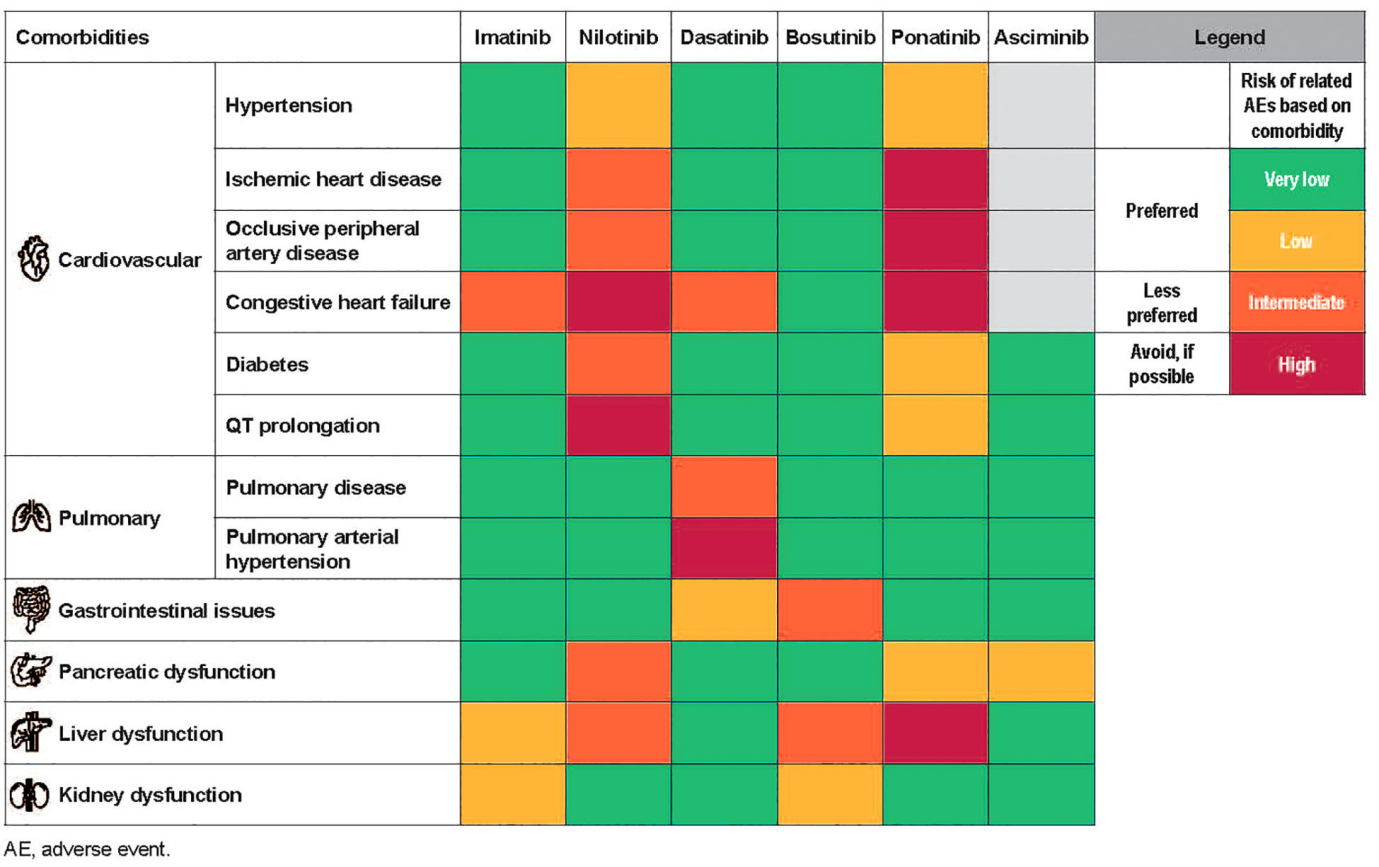
Treatment selection based on comorbidities (17, 70). Each TKI is associated with a unique AE profile which can help guide CML treatment decision-making, particularly in the case of patient comorbidities.

In general, for older patients or those with significant risk factors for AEs due to underlying comorbidities, imatinib tends to be the preferred 1L treatment option (2, 3). For lower-risk patients, imatinib may offer a balance of efficacy and tolerability and be sufficient (2, 3). For patients with higher-risk disease, treatment decision-making becomes more complicated, particularly for those patients with comorbidities (2, 3). Currently, 2G TKIs are preferred in these patients, but selecting which agent is best can be a challenge (2, 3). While there are no formal guidelines, experience with each of the TKIs has allowed us to make some recommendations on treatment selection across all lines of therapy in patients with significant baseline comorbidities (**Figure 2**) (17, 70).

Many physicians find that concerns over potential CV toxicities are some of the most challenging to address when selecting treatment. In a study evaluating incidences of CVEs in 531 patients with newly diagnosed CML-CP from frontline clinical trials, the overall occurrence of CVEs was highest with ponatinib (63%) and similar among the other TKIs (imatinib 400 mg, 49%; imatinib 800 mg, 44%; nilotinib, 41%; dasatinib, 39%; *P*=0.13) (71). Patients with CML have higher baseline CV risk factors, yet conclusions on CV toxicity are difficult to make from clinical trial data due to exclusion of high-risk patients; unsurprisingly, current real-world evidence suggests that patients with high CV risk scores are at the most risk for CVEs (2, 3, 72).

If a treatment switch is needed due to tolerability issues, better tolerability with the next line of therapy is not guaranteed. Before switching, it is important for HCPs to review the most common adverse effects of each TKI with their patients, noting that some of the AEs that prompted the switch may also be seen with any of the other TKIs. It is also essential to cross-check toxicities with patient comorbidities when making a switch due to intolerance. In a patient with a good response at the time of switching, it might be reasonable to consider starting the patient at a low dose and slowly escalating the dose based on tolerance, rather than immediately starting at the full dose. More research is needed to help HCPs manage TKI intolerance and optimize treatment sequencing based on intolerance.

### 4.7 Patient adherence

Patient adherence to therapy also plays a significant role in response (73–78). Several small studies suggest that higher adherence correlates with improved clinical outcomes, including likelihood of achieving molecular response, depth of response, and survival; however, large, randomized studies are lacking. Studies differ with respect to the agents that have higher rates of nonadherence, but the overall rate is approximately 20% to 30% (74, 75, 79). Patients with lower rates of adherence have reduced likelihood of achieving MMR and event-free survival at 5 years compared with patients with higher rates of adherence (80). Patients experiencing poor QOL may be less likely to adhere to their medication. In a Polish study assessing treatment adherence in 140 adults with CML treated with imatinib, dasatinib, or nilotinib, TKI side effects and worry over TKI side effects were cited as reasons for missing a TKI dose in 17.6% and 5.9% of patients, respectively (81). The reasons for nonadherence are multifactorial and may change over time; however, tolerability issues, such as gastrointestinal toxicities and financial concerns, are often the most common contributors (73, 74, 82). Also, medication adherence tends to decrease over time as patients are on treatment for many years (73–78). Adherence with the treatment dosing and administration requirements can also be challenging. For example, nilotinib requires twice-daily dosing in a fasting state. Patients may find these requirements difficult to adhere to on a day-to-day basis as they try to live their lives as normally as possible.

Since adherence is associated with better outcomes, it is important that HCPs managing patients with CML evaluate TKI adherence periodically to help reinforce the importance of taking treatment as directed and to identify strategies to overcome barriers to adherence (2, 3). Since the challenges vary between patients, interventions to address them must also be individualized. More frequent molecular monitoring may be warranted if concerns exist over a patient’s adherence (3).

## 5 Sequencing therapy: 3L and beyond

Patients on 2L+ TKI therapy who do not achieve cytogenetic or molecular responses at 3, 6, or 12 months should be considered for alternative therapies or allo-HCT (2, 3). Guidelines do not include a formalized definition of an acceptable response to 2L+ TKI therapy, although the previous response milestones may be used as a guide (2, 3). The likelihood and durability of response decreases when a different 2G TKI is used after treatment with ≥2 other TKIs (2, 3). Consideration of allo-HCT increases as other treatment options are exhausted (2, 3).

As with switching from 1L to 2L treatment, the decision to switch from 2L to 3L treatment must be individualized based on each patient’s goals and factors related to their overall health and disease (2, 3). In the later-line settings, patients tend to be older with more comorbidities and concurrent medications that may play a role in treatment selection. Outside of the TKIs recommended as 1L and 2L therapy, limited additional options are approved or recommended for use after a 2L TKI. As a result, physicians may be reluctant to switch treatment in the absence of clear progression or loss of response. **Table 4** summarizes data from key later-line studies. Response-adjusted ponatinib dosing (starting dose 45 mg QD, with reduction to 15 mg QD when *BCR::ABL1* ^IS^ ≤1% is achieved) has been FDA approved for patients with resistance to or intolerance of ≥2 TKIs (3). As mentioned previously, the use of ponatinib carries an increased risk of CVEs, particularly arterial occlusive events; therefore, patients must be screened for CV risk factors, which must be controlled before starting therapy with ponatinib (3). In the US, omacetaxine, a protein translation inhibitor, is another later-line treatment option that is indicated for adults with CML-CP, also approved in 2012 for patients with resistance to or intolerance of >2 TKIs. Omacetaxine acts independently of direct BCR::ABL1 binding but has been shown to reduce levels of BCR::ABL1 and Mcl-1 and has shown activity in wild-type and T315I-mutated CML (3, 83, 84).

Asciminib was recently approved in the US for patients with CML-CP who are resistant to or intolerant of ≥2 TKIs based on the results of the phase 3 ASCEMBL study, which demonstrated the efficacy and safety of asciminib versus bosutinib (**Table 5**) (53). The week 96 follow-up from this study continued to show that asciminib was significantly more effective than bosutinib and had a favorable tolerability profile (53). The availability of asciminib gives patients another viable treatment option to further delay the need for transplant and is a particularly important option for those patients who are ineligible for transplant.

As previously described, treatment options for patients with a T315I mutation are limited. Ponatinib and asciminib are approved in the US for the treatment of patients with an acquired T315I mutation (3). As with earlier lines of therapy, ponatinib must be used with caution in patients with CV risk factors. Asciminib is also effective in this setting and, based on current clinical data, may be better tolerated than ponatinib with fewer limitations for use in patients with CV risk factors.

## 6 Newer treatment landscape developments

The treatment landscape for CML-CP continues to evolve since imatinib’s approval in 2001, with additional therapies in development at the time of this writing (66).

### 6.1 Therapies approved in a single country

Flumatinib is a 2G TKI approved in China since 2019 for 1L treatment in patients with CML-CP (85–88). It has demonstrated higher selectivity and potency against BCR::ABL1 compared with imatinib. In the phase 3 FESTnd study conducted in China in newly diagnosed patients with CML-CP, flumatinib demonstrated significantly higher efficacy and lower rates of disease progression vs imatinib (NCT02204644) (87, 89). A study in China is evaluating the efficacy and safety of flumatinib in newly diagnosed CML-CP (NCT04591197) (90). EMR, with continued improvements in molecular response were observed over 12 months (85). A phase 4 dose-optimization study comparing the EMR with flumatinib 400 and 600 mg at 3 months is ongoing in China (NCT05353205) (91).

Radotinib, a 2G BCR::ABL1 inhibitor, was initially approved in South Korea in 2012 for 2L treatment of patients with CML-CP who had insufficient response to prior TKIs (92, 93). Following the higher rates of molecular response with radotinib than imatinib in the RERISE study, radotinib was approved in South Korea for 1L treatment in 2015 (NCT01511289) (93, 94). Currently, a multinational, phase 3 study of radotinib in patients with CML-CP who experienced treatment failure or intolerance of previous TKIs is evaluating major cytogenetic response at 6 months (NCT03459534) (95).

Olverembatinib is a third-generation ATP-competitive TKI approved in China in 2021 for the treatment of TKI-resistant CML-CP or -accelerated phase harboring the T315I mutation (96). The efficacy results of 2 clinical studies conducted in China are reported in the 2024 NCCN Clinical Practice Guidelines in Oncology in CML (NCT03883087 and NCT03883100) (97, 98). A phase 3 study is being conducted in China in patients with CML-CP who have resistance to and/or intolerance of ≥2 2G TKIs to evaluate MMR at 12 months (NCT05311943) (99).

### 6.2 Investigational therapies

Vodobatinib, a third-generation ATP-competitive TKI, is being evaluated in a two-part phase 1/2 study (NCT02629692) (100–102). Patients with CML-CP who experienced treatment failure with ≥3 TKIs received escalating doses of vodobatinib ranging from 12 to 240 mg. The ongoing phase 2 study evaluates vodobatinib at the recommended phase 2 dose of 174 mg once daily in patients with CML-CP experiencing treatment failure with ≥3 prior TKIs or less (if not eligible for other approved 3G TKIs) (100, 103).

ELVN-001 is a next generation ATP-competitive TKI currently being tested in a phase 1a/1b dose-escalation study in patients with CML-CP with and without the T315I mutation to evaluate efficacy, safety, and tolerability (NCT05304377) (104, 105).

TERN-701, an allosteric inhibitor that targets the ABL myristoyl pocket, is in a phase 1 dose-escalation study in China to determine the maximum tolerated dose (NCT05367700) (106, 107). The phase 1 CARDINAL study of TERN-701 was initiated in the United States in patients with CML-CP who experienced treatment failure, suboptimal response, or intolerance to prior TKI treatments (NCT06163430). Part 1 will evaluate sequential dose-escalation cohorts of TERN-701 administered once daily and part 2 is a randomized, parallel dose (100–102) expansion cohort of ≥2 recommended dose levels from part 1 (106). The FDA granted TERN-701 an orphan drug designation for the treatment of CML in March 2024 (108).

AS1266, an allosteric TKI that binds to the myristoyl pocket of the BCR::ABL fusion protein, is currently in the pre-clinical stage of development (109, 110).

## 7 Conclusions

The availability of more treatment options has significantly prolonged the lives of patients with CML-CP and given physicians the option to sequence these therapies to optimize outcomes and help patients achieve their treatment goals. However, definitive guidelines on how to most effectively sequence therapies are lacking, and physicians need to weigh numerous factors when making these treatment decisions. Additional therapies are in development, and ongoing research on currently approved TKIs will help improve decisions around treatment selection and sequencing. With additional therapies in development, later-line treatments for CML are evolving. As more options become available and more patients are receiving ≥3 lines of therapy, we must continue to evolve the goals of therapy and optimize treatment sequencing to ensure that all patients attain the best possible outcome for their situation.

## Author contributions

DA: Writing – original draft, Writing – review & editing. VK: Writing – original draft, Writing – review & editing. KS: Writing – original draft, Writing – review & editing.
